# Specific fibre areas in the femoral footprint of the posterior cruciate ligament act as a major contributor in resisting posterior tibial displacement: A biomechanical robotic investigation

**DOI:** 10.1002/ksa.12486

**Published:** 2024-09-26

**Authors:** Adrian Deichsel, Thorben Briese, Wenke Liu, Michael J. Raschke, Alina Albert, Christian Peez, Andreas Weiler, Christoph Kittl

**Affiliations:** ^1^ Department of Trauma, Hand and Reconstructive Surgery University Hospital Münster Münster Germany; ^2^ Sporthopaedicum Berlin Berlin Germany

**Keywords:** biomechanics, direct fibres, PCL, posterior cruciate ligament, reconstruction

## Abstract

**Purpose:**

Similar to the anterior cruciate ligament, the femoral footprint of the posterior cruciate ligament (PCL) is composed of different fibre areas, possibly having distinct biomechanical functions. The aim of this study was to determine the role of different fibre areas of the femoral footprint of the PCL in restraining posterior tibial translation (PTT).

**Methods:**

A sequential cutting study was performed on eight fresh‐frozen human knee specimens, utilizing a six‐degrees‐of‐freedom robotic test setup. The femoral attachment of the PCL was divided into 15 areas, which were sequentially cut from the bone in a randomized sequence. After determining the native knee kinematics, a displacement‐controlled protocol was performed replaying the native motion, while constantly measuring the force. The reduction of the restraining force presented the percentage contribution of each cut, according to the principle of superposition.

**Results:**

The PCL was found to contribute 29 ± 16% in 0°, 51 ± 24% in 30°, 60 ± 22% in 60° and 55 ± 18% in 90°, to restricting a PTT. The fibre areas contributing the most were located at the proximal border of the PCL footprint, away from the cartilage, and directly adjacent to the medial intercondylar ridge (*p *< 0.05). Of these, one fibre area showed the highest contribution at all flexion angles. This area was located at the posterior half of the medial intercondylar ridge. No clear assignment of the areas to either the anterolateral or posteromedial bundle was possible.

**Conclusion:**

An area towards the proximal and posterior part of the femoral PCL footprint was found to significantly restrain a posterior tibial force. Based on the data of this testing setup, a PCL graft positioned at the identified area may best mimic the part of the native PCL, which bears the most load in resisting a PTT force.

**Level of Evidence:**

No evidence level (laboratory study).

**Abbreviations**
ACL: anterior cruciate ligament
ALB: anterolateral bundle
aMFL: anterior meniscofemoral ligament
ATT: anterior tibial translation
CI: confidence Interval
DOF: degrees of freedom
MD: mean difference
PCL: posterior cruciate ligament
PCLR: posterior cruciate ligament reconstruction
PMB: posteromedial bundle
pMFL: posterior meniscofemoral ligament
PTT: posterior tibial translation

## INTRODUCTION

The posterior cruciate ligament (PCL) is typically described as a two‐bundle structure, consisting of the anterolateral bundle (ALB) and the posteromedial bundle (PMB) [6]. The ALB is described to be more active in early flexion, while the PMB is described to be more active in deep flexion [25]. However, no clear dominance of a bundle in different flexion angles is evident and insufficiency of both bundles has to be present to result in clinically perceivable posterior instability [15, 25].

To restore the physiological biomechanical functions in a PCL‐deficient knee, a PCL reconstruction (PCLR) with a graft mimicking the native restraint would be favourable, to prevent osteoarthritis and preserve sporting activity [25, 26, 29, 34]. However, the optimal femoral and tibial tunnel positions for PCLR are still controversially debated. Due to the large surface of the femoral footprint, different tunnel positions are possible, including single‐bundle ALB, and PMB reconstructions, isometric reconstructions and double‐bundle reconstructions, with a wide array of possible surgical techniques [26, 35]. For the anterior cruciate ligament (ACL), specific fibre areas of the femoral and tibial footprint were biomechanically shown to be the primary contributors in restraining an anterior tibial translation (ATT) force, informing the clinician on where to position the femoral tunnel [14, 18, 28]. Analogous, the femoral footprint of the PCL is also comprised of discernable fibre areas, which might possibly show different contributions to restrain posterior tibial translation (PTT) [4, 28].

The purpose of this study was to investigate the different fibre areas of the femoral footprint of the PCL regarding their contribution to resisting a PTT force. It was hypothesized, corresponding to the biomechanical study of the ACL [14], that the area close to the medial intercondylar ridge will bear more load than the fibre areas towards the cartilage border.

## MATERIALS AND METHODS

The experiments were performed with permission from the Institutional Review Board of the University of Münster (IRB reference number 2023‐407‐f‐S). Eight unpaired cadaveric knee specimens (mean age 67.9 ± 7.7 years) without prior knee surgery were obtained from MedCure. The specimens were visually inspected for high‐grade osteoarthritis (Kellgren–Lawrence score >2), or meniscal injury. Furthermore, ligamentous instability testing was performed by the investigator. Specimens were excluded if pathologies were present.

Specimens were stored at –20°C and thawed for 24 h at room temperature, prior to preparation. The skin and subcutaneous fat were resected, leaving the rest of the soft tissues intact. Tibia and femur were secured in aluminium cylinders, 12 cm above and below the joint line, with three‐component polyurethane bone cement (RenCast®; Gößl & Pfaff). The fibula was then cut 10 cm below the joint line and transfixed with a 3.5 mm cortical screw to the tibia. According to the descriptions of Merican et al., a longitudinal transpatellar osteotomy was performed, to allow visualization and subsequent cutting of the femoral PCL attachment [22]. Specimens were wrapped in wet tissue papers to prevent drying.

### Robotic test setup

**Figure 1:**
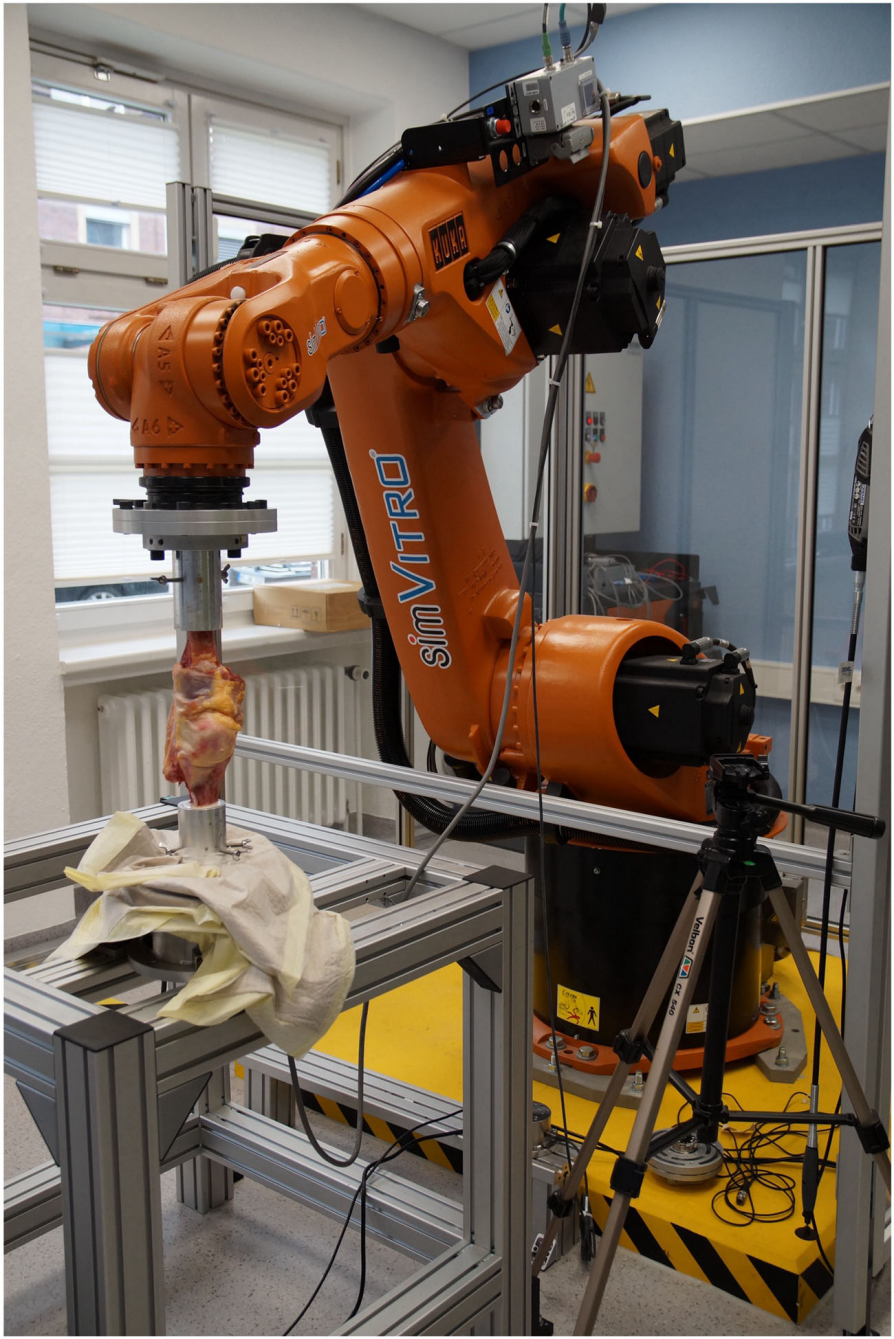
Robotic six‐degrees‐of‐freedom robotic test setup.

A validated setup consisting of six‐degrees‐of‐freedom (DOF) industrial robot (KR 60‐3; KUKA Robotics) equipped with a force–torque sensor (FTI Theta; ATI Industrial Automation) was used for biomechanical testing in this study (Figure 1), as previously described [1, 8]. The robotic system allows for position‐controlled movement with an accuracy of ±0.06 mm, as well as force‐controlled movement with an accuracy of ±0.25 N and ±0.05 Nm, respectively. The test setup was driven by the custom software simVITRO (Cleveland Clinic BioRobotics Lab), which optimizes the robot test setup for the simulation and acquisition of knee joint kinematics. The knee joint coordinate system was defined according to the descriptions by Grood and Suntay [9], by digitizing landmarks on the femur and tibia, using a tactile measuring arm (Absolute Arm 8320‐7; Hexagon Metrology GmbH), which has an accuracy of ±0.05 mm. Translations and rotations of the tibia in relation to the femur and the corresponding forces/torques in all six DOF were recorded continuously.

### Biomechanical testing

To minimize tissue hysteresis, each specimen was flexed and extended 10 times. The starting point of each knee was determined by manually minimizing all forces and torques acting on the knee in full extension. The passive path of the knee was then determined by flexing each knee from full extension to 90° of flexion while minimizing forces and torques in all axes aside from the flexion‐extension axis. An axial compression force of 50 N was applied to warrant contact between the femur and tibia during the passive path. A force‐controlled testing protocol (recording displacements in response to given forces/torques), applying 89 N ATT force, and 89 N PTT force, was performed in 0°, 30°, 60° and 90° of flexion under axial compression of 200 N. The displacements during the force‐controlled cycle were continuously recorded. The motion of the native knee was then transferred to a displacement‐controlled test protocol (recording forces in response to given displacements). The reduction of force after applying the simulated laxity tests indicated the contribution of each cut in restricting ATT/PTT [2, 27].

### Sequential cutting protocol

**Figure 2:**
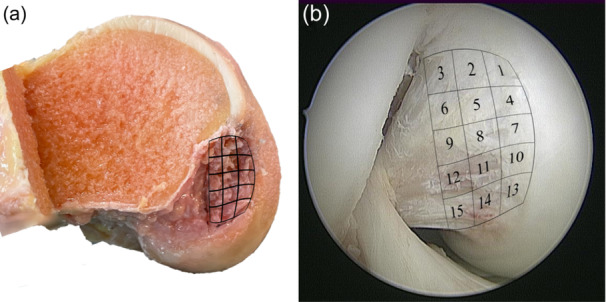
Grid depicting sequential cutting steps of the femoral footprint of the posterior cruciate ligament. (a) Anatomical view of the medial notch wall. (b) Arthroscopic view of the medial notch wall. The femoral footprint was divided into 15 areas arranged in five rows, and three columns. Cutting was performed from distal (towards the cartilage) to proximal (away from the cartilage border), starting either at the most ventral row (1–3) or most dorsal row (13–15).

First, the native knee kinematics were recorded, utilizing the displacement‐controlled test protocol. The anterior meniscofemoral ligament (aMFL), and posterior meniscofemoral ligament (pMFL), connecting the lateral meniscus to the medial femoral condyle were cut to exclude their influence on the sequential cutting of the femoral footprint of the PCL. Before starting sequential cutting, the dimensions of the femoral footprint of the PCL on the medial notch wall was measured using the tactile measuring arm (Absolute Arm 8320‐7; Hexagon Metrology GmbH). Based on the measurements, a grid was calculated dividing the femoral footprint, containing the aMFL, pMFL, ALB and PMB, into 15 areas (Figure 2). In each subsequent cutting step, a single area was cut to determine its contribution to resisting knee motion in the investigated directions. By cutting different areas inside the PCL, the first principle of superposition was violated [27, 37]. To account for this shortcoming, the cutting sequence was randomized. Cutting was performed starting either with the most ventral (Numbers 1, 2, 3) or most dorsal row (Numbers 13, 14, 15) of areas in a randomized fashion, to minimize the effect of a potential load‐sharing effect across the femoral footprint of the PCL [14, 18].

### Quantification of the medial notch wall

After biomechanical testing, each tested knee was exarticulated and the peripheral soft tissues at the femur were completely resected. The lateral femoral condyle was excised, by splitting the femur through the deepest part of the trochlea, to gain full visualization of the medial notch wall. The surface of the femoral footprint of the PCL, as well as the medial notch wall was digitized using the tactile measuring arm. Calculation of the dimensions and surfaces were performed using the PC‐DMIS software (Hexagon Metrology GmbH).

### Data analysis, statistics and sample size calculation

Extraction of knee kinematics from the raw data of SimVitro was performed using Matlab (version R2020a, MathWorks), and Excel (Microsoft). Statistical analysis was performed using PRISM (version 8, GraphPad Software). The contribution of the PCL to restraining PTT is presented as a percentage of the total forces determined in the native state (89 N). The contribution of each area of the femoral PCL footprint is presented as a percentage of the contribution of the total PCL. The loss of force during the application of an ATT force served as an internal control. The comparison of the total contribution of the PCL in different flexion angles was performed utilizing a repeated‐measures one‐way ANOVA. Subsequently, mixed linear models were used to assess the main effects and interactions of each independent variable (cutting state in different flexion angles), with the dependent variable being the PTT. Post hoc pairwise comparisons were used to compare the contribution of each cutting state against the intact PCL state. The results are presented as mean differences (MD) with corresponding 95% confidence intervals (CI). Post hoc Dunn's correction was performed to account for multiple testing. A *p*‐value less than 0.05 was deemed to identify statistically significant differences. To investigate the effect of the cutting sequence, in the cutting steps that were statistically significant contributors, the influence of the cutting sequence was assessed using mixed linear models with the flexion angle and the type of cutting sequence (starting either from ventral or dorsal) as the independent variables.

To calculate the sample size necessary for the present study, an a priori power analysis was performed using G*Power (version 3.1) [7]. Based on previous studies investigating the influence of different fibre bundles on the in situ forces of cruciate ligaments [14, 18], a sample size of *n* = 8 was calculated to show a 10% contribution of a cutting step (assuming a standard deviation of 10%; effect size = 1), with a power of 80%, at the significance level of *p *< 0.05.

## RESULTS

No specimen had to be excluded, resulting in every tested knee being available for final analysis. An aMFL was present in 5/8 tested specimens and a pMFL in 4/8 specimens. Both aMFL and pMFL were not found to be statistically significant contributors to the restriction of a PTT force (*p *> 0.05).

The dimensions of the grid laid over the femoral footprint of the PCL had a mean length of 29.2 ± 3.1 mm and a mean width of 14.1 ± 2 mm. The area of the femoral footprint of the PCL was 340.3 ± 78.1 mm^2^. The surface of the medial femoral notch wall covered by the PCL footprint was 42.2 ± 6.7%.

### Contribution of the total PCL to the restraint of PTT in different flexion angles

**Figure 3:**
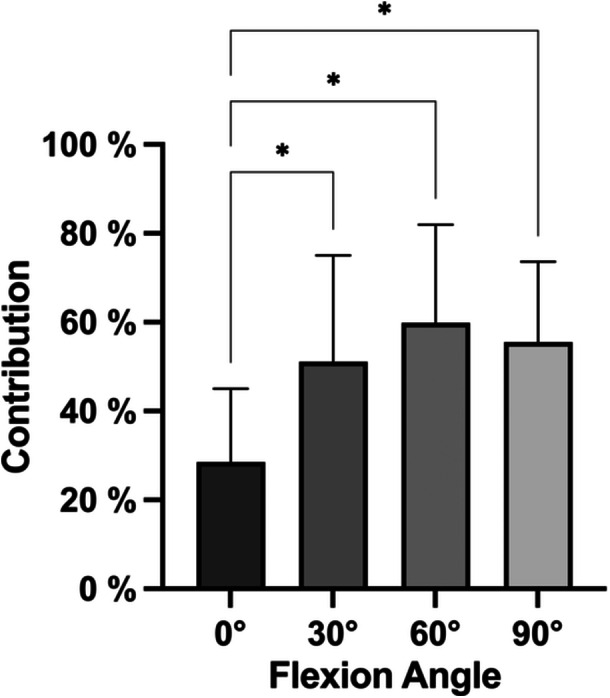
Relative contribution of the total posterior cruciate ligament (PCL) to restricting posterior tibial translation in different flexion angles. *p < 0.05.

The complete PCL was found to contribute 29 ± 16% in 0° of flexion, 51 ± 24% in 30° of flexion, 60 ± 22% in 60° of flexion and 55 ± 18% in 90° of flexion. The contribution of the PCL to restricting PTT was found to be significantly (*p *≤ 0.05) lower in 0° of flexion, compared to 30°, 60° and 90° (Figure 3).

### Sequential cutting of the femoral footprint of the PCL

**Figure 4:**
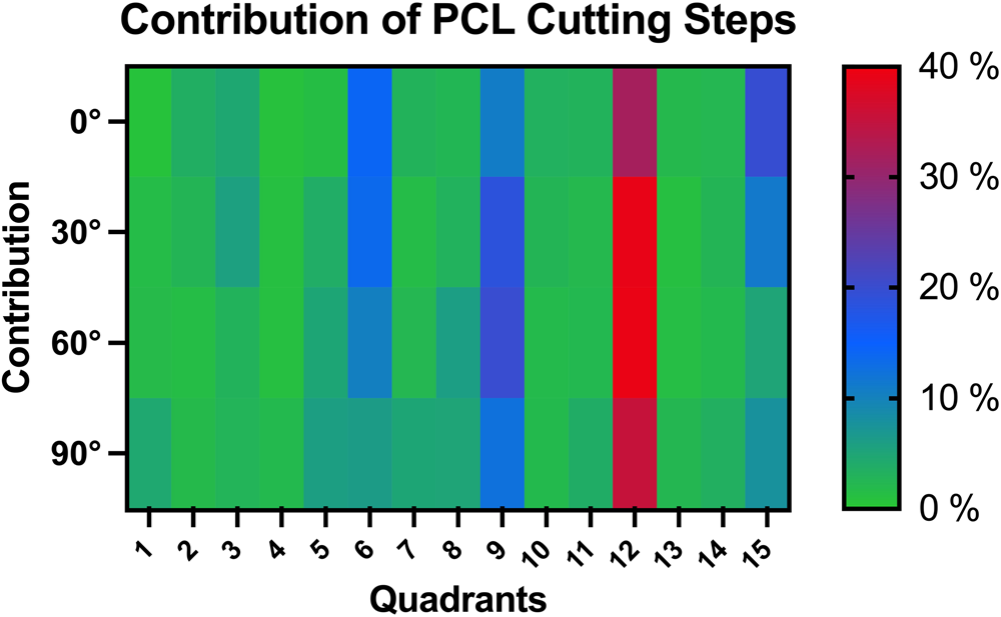
Heatmap visualization of the contribution of each cutting step of the femoral footprint of the posterior cruciate ligament (PCL) on restricting posterior tibial translation in different flexion angles. The contribution of each cutting step is presented as the mean percentage of the contribution of the total PCL.

It was found that the major contributors (>10% contribution) to the PCL's ability to restrain PTT force were located at the proximal border of the PCL footprint, away from the cartilage border, and directly adjacent to the medial intercondylar ridge (Figures 2 and 4).

**Figure 5:**
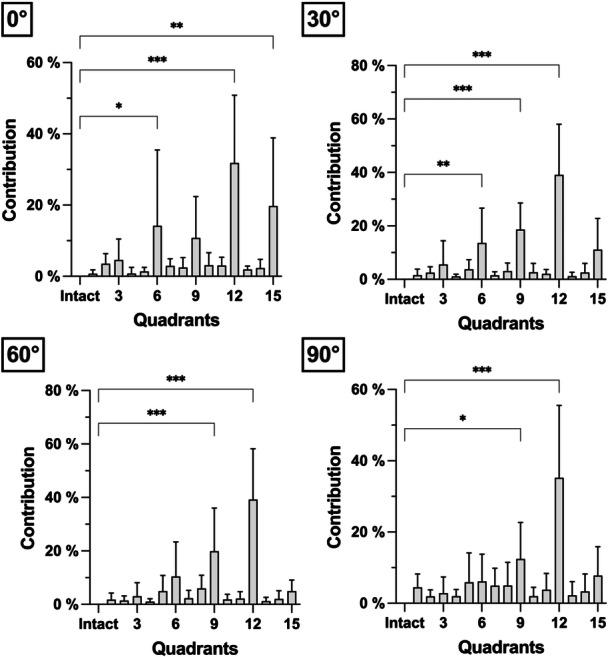
Relative contribution of the cutting steps to the restraining force of the posterior cruciate ligament in different flexion angles. Multiple comparisons were performed against the posterior cruciate ligament intact state; * *p *< 0.05; **p < 0.01; ***p < 0.001.

Areas 6, 9, 12 and 15 were shown to be statistically significant contributors to restraint of PTT force (Table 1 and Figure 5). Of these, area 12 constantly showed the highest contribution (≥30%) across all flexion angles. Together, areas 6, 9 and 12 contributed 52.2%–75.9% from 0° to 90° of flexion. No clear assignment of the areas to either the anterolateral or posteromedial bundle was possible.

**Table 1:** Fibre bundles significantly contributing to the posterior cruciate ligaments ability to restrain a posterior tibial translation force.

Flexion angle	Cutting step	Contribution (mean + 95% confidence interval)	*p* Value
0°	6	13% (1%–25%)	0.04
	12	31% (19%–44%)	<0.001
	15	19% (7%–32%)	0.001
30°	6	14% (2%–25%)	0.004
	9	19% (7%–30%)	<0.001
	12	38% (26%–50%)	<0.001
60°	9	20% (8%–32%)	<0.001
	12	39% (27%– 51%)	<0.001
90°	9	12% (1%– 24%)	0.01
	12	35% (23%– 47%)	<0.001

### Effect of PCL cutting on ATT force

Sequentially cutting the PCL did not significantly reduce ATT force (*p *> 0.05). This indicates that drops in the contributing forces are only caused by the cutting sequence and are not random.

### Influence of the cutting sequence

No statistically significant difference was found between a cutting sequence starting either from ventral (area 1) or dorsal (area 13; *p *> 0.05).

## DISCUSSION

The most important finding of this study was that specific fibre areas of the femoral footprint of the PCL contributed a major part (>50%) to the ability of the PCL to restrain PTT force. The respective areas were located towards the proximal and dorsal (deep) part of the femoral PCL footprint, away from the cartilage border, corresponding approximately to the 3 o'clock position (in the right knee, 9 o'clock in the left knee) [6]. Verifying the present study's hypothesis, these fibre areas were located in close proximity to the medial intercondylar ridge corresponding to the direct fibre insertions [4].

Previous biomechanical studies focused mostly on the function of the ALB and PMB, when investigating the role of the PCL in restraining PTT. An early study investigating the effect of cutting the PCL, using a material testing machine, found that the ALB was the main contributor to restricting the PTT from 40° to 120° (contribution of 40%–74%), while the PMB became more active in deep flexion (up to 57% contribution in 130° of flexion) [25]. In full extension, 65%–73% of a PTT was restricted by structures other than the PCL, which is in accordance with the results of the present study with the PCL only contributing 29 ± 16% to restraining a PTT force, in full extension. Another biomechanical study, utilizing a robotic testing system to investigate the functions of the ALB and PMB, found that isolated sectioning of these bundles led to a statistically significant, but small increase in PTT (0.9 ± 0.6 mm for the ALB and 2.6 ± 1.8 mm for the PMB in 90° of flexion) [15]. Sectioning of the whole PCL led to a higher increase of 11.7 ± 4.0 mm in PTT. An explanation for these findings could be that in force‐controlled sequential cutting studies, the last cut of a structure has the largest effect, while partial cutting only has minor effects [16, 20]. This may also be related to the findings of the present study, that the most load‐bearing areas were located in both bundles (area 6—ALB; area 15—PMB) and at the intersection between those (areas 9 and 12) [8].

The results of the present study are explainable by investigating the composition of the PCL fibres inserting into its footprint on the medial femoral notch wall. Previously, ligaments as well as tendons were shown to insert into the bone by either direct or indirect insertions, with direct fibres assumed to be the main transmitters of force [32]. Regarding the femoral footprint of the ACL, direct fibre areas close to the lateral intercondylar ridge were found to be the main contributor (82%−90%) in restraining an ATT force in a biomechanical study [14, 28]. In a recent histo‐anatomical study, it was shown that direct fibres in the femoral footprint of the PCL were located at the proximal (deep) border of the footprint (which was located a mean of 16.5 mm from the anterior cartilage border), directly adjacent to the medical intercondylar ridge [4]. These locations of the direct fibres directly correlate with the locations of the areas contributing the major part to the in situ forces of the PCL in the present study. Furthermore, this area corresponds to the descriptions of multiple authors, describing the PCL as a flat, ‘ribbon‐like’ structure [19, 21], similar to the ACL [13, 30].

The present study is of clinical relevance due to its implications for clinical practice. The failure rate of PCLR is reported to be up to 18% [5, 24, 31]. A suboptimal positioning of the femoral bone tunnel may be one reason for the clinical failure of PCLR, as it might lead to unphysiological forces acting on the reconstruction over the flexion‐extension cycle [3, 10]. Several techniques for PCLR are published, including single‐bundle reconstructions, double‐bundle reconstructions and isometric single‐bundle reconstructions. Biomechanical studies generally found the double‐bundle reconstruction to reduce the posterior translation of the tibia more effectively than a single‐bundle reconstruction [11, 33]. However, in most studies, single‐bundle PCLR was performed by isolated replacement of the ALB. Furthermore, the clinical superiority of a double‐bundle PCLR, in comparison to a single‐bundle PCLR could not be clearly established [17], leading many surgeons to perform single‐bundle reconstructions, mostly in the ALB position [36]. Based on the findings of the present study a single‐bundle PCLR of either the ALB or PMB does not seem best to mimic the natural restraint of the PCL's ability to restrict PTT, since the fibre areas with the highest contribution are located both in the ALB and PMB. Alternatively, an isometric PCLR, defined as a reconstruction with minimal length change over the flexion‐extension cycle, can be performed. The isometric point, as defined by multiple authors, is located close to the areas of the highest statistically significant contribution (areas 9 + 12) in the present study [26]. However, isometric reconstructions were not able to completely restore PTT to the native state, which might be explainable by a round bone tunnel not being able to encompass enough surface of the significant contributors [26]. In summary, based on the results of the present study, a PCLR reconstruction mimicking the significantly contributing areas of the femoral PCL footprint may best imitate the load‐bearing areas of the native PCL regarding restraint of a PTT force. This might be realized by performing a flat or rectangular reconstruction.

Several limitations have to be considered when interpreting the results of the present study.

In previous anatomical studies of the ACL, a change in the morphology of the tibial and femoral insertion sites was described, with older specimens (≥50 years of age) [23]. Whether this is the case for the PCL is unclear. Very small areas were dissected during sequential cutting of the femoral footprint. Although performed after meticulous planning, it is possible that cuts were performed either larger or smaller than intended. Furthermore, by cutting different areas of the PCL, the first principle of superposition has been violated [27, 37]. To account for this shortcoming, the cutting sequence was randomized. Furthermore, in studies investigating different fibre areas of a single ligament, superposition was used as well [12, 14, 18].

## CONCLUSION

An area towards the proximal and posterior part of the femoral PCL footprint was found to significantly restrain a posterior tibial drawer force. Based on the data of this testing setup, a PCL graft positioned at the identified area may best mimic the part of the native PCL, which bears the most load in resisting a PTT force.

## AUTHOR CONTRIBUTIONS

**Adrian Deichsel**: Conception and design; testing and data acquisition; statistical analysis; writing; proofreading. **Thorben Briese**: Internal review; testing and data acquisition; statistical analysis; writing; proofreading. **Wenke Liu**: Testing and data acquisition. **Michael J. Raschke**: Supervision; internal review; proofreading. **Alina Albert**: Testing and data acquisition. **Christian Peez**: Internal review; proofreading. **Andreas Weiler**: Internal review; proofreading. **Christoph Kittl**: Conception and design; internal review, proofreading.

## CONFLICT OF INTEREST STATEMENT

Adrian Deichsel is Web Editor for Knee Surgery, Sports Traumatology and Arthroscopy (KSSTA). The remaining authors declare no conflict of interest.

## ETHICS STATEMENT

The specimens were dissected and biomechanically tested under the approval of the Institutional Ethics Committee of the University of Muenster (File number 2023‐407‐f‐S).

## Data Availability

Data are available from the corresponding author upon reasonable request.
